# Fatemeh Makkizadeh Ph.D., Esmaeil Bigdeloo M.A.

**DOI:** 10.18502/ijrm.v17i5.4602

**Published:** 2019-06-26

**Authors:** Fatemeh Makkizadeh, Esmaeil Bigdeloo

**Affiliations:** School of Social Sciences, Yazd University, Yazd, Iran.

**Keywords:** Andrology, Co-word analysis, Intellectual structure.

## Abstract

**Background:**

The Co-word analysis has the ability to identify the intellectual structure of knowledge in a research domain and reveal its subsurface research aspects.

**Objective:**

This study examines the intellectual structure of knowledge in the field of Andrology during the period 2008-2017 using Co-word analysis.

**Materials and Methods:**

In this descriptive-analytical study with a scientometric approach, the WoS database was searched for papers indexed under “Andrology” over the period 2008–2017. The data were analyzed using Co-word, clustering methods, and strategic diagram with the help of SPSS, UcInet, RavarPreMap and VOSviewer software.

**Results:**

The highest publication rate in the area of Andrology was seen in countries like the USA, China, Italy, and Iran. The top three journals that published papers on the field were Fertility and Sterility, Andrologia, Human reproduction. The results showed that the keyword “Spermatozoa” and two pairs of frequently used keywords, namely “Azoospermia * Oligospermia” were the most frequent in the field of Andrology. The results of hierarchical clustering led to 13 clusters. The clusters “Reproductive Techniques” and “Spermatogenesis” are the core clusters and play an effective role. The "Post-Testicular causes” and “Neoplasm” clusters are in marginal.

**Conclusion:**

This study represented that Co-word analysis can well illustrate the intellectual structure of an area. Considering the frequency of keywords along with the clusters obtained, it seems that the majority of research approach was seen on infertility treatments, especially through assisted reproductive technology. Despite the importance of psychological aspects as well as education of reproductive health, these subjects have not been sufficiently considered.

## 1. Introduction

“Andrology is a scientific or medical discipline concerning the study of male reproductive biology, diseases of the male genital organs, and male infertility under physiological and pathological conditions. If this definition were to be interpreted in the context of sociobiology considering reproduction, the central task of life to which the entire organism is devoted, andrology would then be a broad field” (1). Andrology has merely been studied since the late 1960s (2). Male infertility is a multifactorial disorder, which affects about 10% of couples at the age of fertility (3) and about 4-17% of couples seeking medical therapy for infertility. Eventually, 3-4% of all them stay unintended childless at the end of their reproductive life stage (4). In order to obtain clear evidence to cure infertility among males and females, we require to investigate and detect active research fields in this area so that the physicians and scholars can benefit from the fresh knowledge yielded in this manner. This study investigates the researches done on andrology so far in terms of content. It also studies the main topics in researches and reviews thematic relations using the Co-word analysis method. This type of analysis can help us find hidden relations in one area of science, promote a concept during a specific period (5), unveil trends in a certain sphere (6), determine outstanding and significant issues in an area, and discover the topics and concepts that are dominant in the works presented by the researchers (7). Concepts scrutinized in this study are in fact keywords that exist on the Web of Science (WoS) database in researches on Andrology. Each keyword in the investigated texts will be considered as a variable that is calculated through a co-occurrence matrix of each variable (keyword) with other variables (other keywords). Based on such calculation, the variables are divided into different axes in Andrology area via application of clustering technique. Considering the function of Co-word analysis, the current study can scientifically delimit the intellectual structure that governs the Andrology area. Literature review showed that the Co-word analysis has been used in various fields of science, including: integrative and complementary medicine (8), anticancer (9), treatment of depression (10), business (11), electrically conductive nanocomposites (12), nanoscience (13), creativity (14), social media (15), infertility (16), and information behavior (17). Reviewing background shows that the intellectual structure of knowledge and its research front can be identified by the Co-word analysis.

This research seeks to take an analytical approach and attempts to reveal the intellectual structure of knowledge in Andrology on WoS during the period 2008-2017, via Co-word, network analysis, and scientific visualization tools. In addition, this study introduces the countries and journals that produced documents in Andrology area.

## 2. Materials and Methods

We chose WoS database as data source. Web of Science is an online subscription-based scientific citation indexing service originally produced by the Institute of Scientific Information (ISI), that provides a comprehensive citation search. It gives access to multiple databases that reference cross-disciplinary research, which allows for an in-depth exploration of specialized sub-fields within an academic or a scientific discipline.Data includes all the documents that have been published within the Andrology field from 2008 to 2017 in journal indexed in the Web of Science Database. To extract the data the following query was used: ((Topic (TS tag) = (Male AND (infertility OR Sterility OR Sub*fertility or andrology)) AND Topic (TS tag) = (Human*OR Homo sapiens OR Modern Man OR Man OR People))).

After retrieving data, at first, bibliometric analysis of years, keywords and countries that contributed to producing scientific articles on Andrology was done. After that, all the articles were extracted (as many as 3,869 records), and the author keywords were used. In order to avoid the influence of synonymy and polysemy, all the author's keywords were matched with the Medical Subject Headings (MeSH).

In the next stage, as many as 57 keywords with frequencies of more than 24 were identified and selected as keywords based on Bradford's law. Various thresholds for choosing the top keywords, in the final analysis, have been used in other researches that have been done with Co-word analysis (18, 19).

These keywords, as the main concepts, are capable of displaying the main content of the research. After determining the co-occurrence keyword, the symmetrical co-occurrence matrix, by using RavarPreMap software, was created.

In order to perform the Co-word analysis, hierarchical clustering is usually used. Hierarchical clustering was performed (done) using SPSS 20 software. Clustering analysis can show clusters of keywords (topics) and the relations among them. The significance and location of each topic cluster can then be obtained, based on co-word data Further, analyses of network characteristics yielded from co-occurrence matrix using Ucinet6.0 and VOSviewer in the research are as follow:

### Centrality

The measurement of interaction a cluster has with other sections of the network. In a network, if the node has a large number of interconnection with others, it has a higher centrality and stands in a basic status in the network. Centrality is applied to measure the connection degree between various topics. The more powerful links a cluster takes in a network, the more central its situation becomes (20).

### Density

The assessment of a cluster's growth. A higher density means higher internal correlation degree among nodes. The density of a field shows its ability to hold and expand itself. The density provides a good show of a cluster's capability to maintain itself and develop as time goes (20).

**Figure 1:**
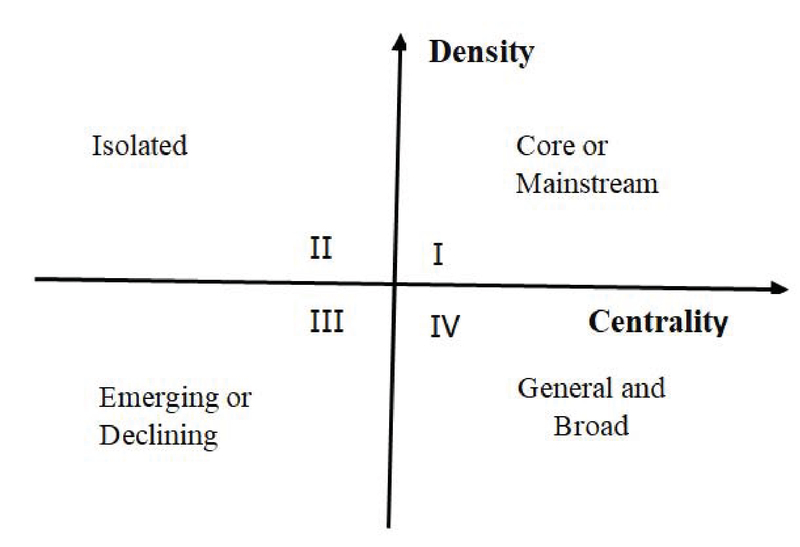
Strategic diagram characterizations based on density and centrality (20).

In a strategic diagram, Greater centrality means that this cluster is highly connected to other clusters and the cluster with higher density means that the keywords within this cluster have a high degree of association with one another. Due to this, in the strategic diagram, y-axis represents density centrality and x-axis represents degree; the average of these two axes is the base (Figure 1).

Different centralities and densities display different status of research topics in four different quadrants. Research topics in first quadrant, display greater centrality and density than the other topics. They are the mainstream issues in research. In second quadrant, research topics are not central but are well-developed. In third quadrant, research topics have lower density and centrality, which means that these research topics have not yet been studied extensively and that they are not highly associated with the others topics. Finally, the research topics in fourth quadrant are highly connected to other topics and occupies an important position in the literature. To understand the current status and trends of Andrology, a strategic diagram calculate the centrality and density of each topics cluster.

### Statistical analysis

Based on the information derived, we used SPSS software V. 20 to perform hierarchical clustering. The centrality and density were obtained by generating Co-word matrix through RavarPreMap and Ucinet6.0. Next, a strategic diagram is drawn to display the current status of research topics on the basis of the centrality and density of each cluster. In addition, VOSviewer software generated a relation network that visualizes the structure keywords. Also Excel was used for the descriptive statistical analysis of the data.

## 3. Results

### Bibliometric analysis

This study collected and analysed 3,891 articles related to Andrology published between 2008–2017 in the WoS database. The top five countries published 52.63% the articles in Andrology field (Table I). 746 different journals published all paper on the field of Andrology during the period 2008–2017. Table II displays the top five journals in the research period.

For getting accurate results, the keywords are standardized. First, we use “Medical Subject Headings (MeSH)” to standardize them. Finally, 57 keywords with a frequency of more than 24 were selected as the research sample for Co-word analysis. These 57 keywords with a total frequency of 4,230 (about 30% of the total) are able to show the main contents of Andrology field in WoS. The top 20 keywords were showed in Table III.

According to Table III, frequently used words include concepts such as Spermatozoa, Azoospermia, Varicocele, Semen Analysis, etc.

**Figure 2:**
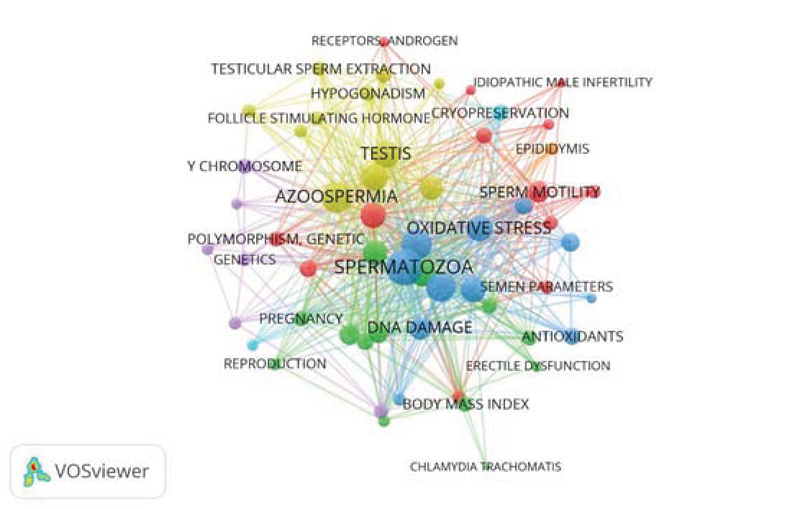
A general overview of co-occurrence Andrology words network over the period 2008-2017.

The structure of the co-occurrence network of the high-frequency words in Andrology articles is shown in Figure 2. In this network, a node represents a high-frequency words and ties represent the relationship between the keywords in all Andrology articles. As it is shown in Figure 2, Since the ties predominantly outnumber the nodes, the mapped network is of a continuous type and is composed of a big network.

After determining the threshold for the coverage of keywords in the Co-word analysis, the rate of the Co-word was obtained. At this stage, the rate Co-word of 57 frequently used keywords with all the keywords in the articles was obtained. The top 20 pairs of frequently used keywords are shown in Table IV.

According to Table IV, the occurrence between the two keywords, “Azoospermia and Oligospermia” is the highest frequency in the field of Andrology. Two pairs of frequently used keywords namely “Spermatozoa”*“Semen” and “DNA Fragmentation”*“Spermatozoa” are ranked second and third respectively. With the aim of visualizing the entire structure of these keywords in the field of andrology, we use the hierarchical clustering approaches, multidimensional scale, and strategic diagrams.

### Multivariate statistical analysis

**Figure 3:**
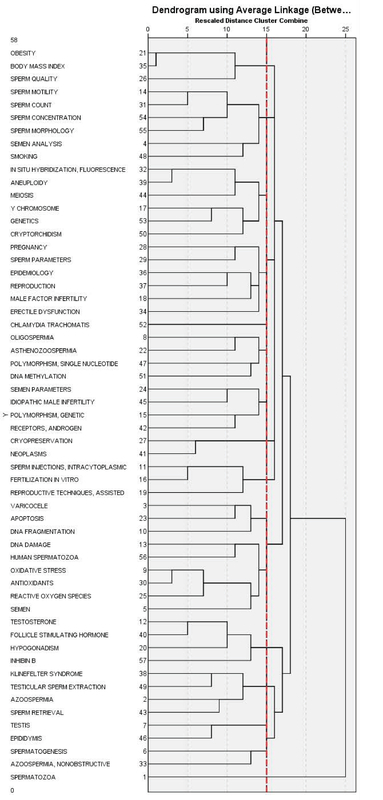
Dendrograms derived from hierarchical clustering by Co-word.

We conducted the cluster analysis using hierarchical clustering with Ward'd method. The first correlation matrix was transferred into SPSS, then the clusters and dendrogram of hierarchical clustering is illustrated (Figure 3).

As the figure shows, the Co-word analysis resulted in 13 clusters. It should be noted that in some clusters, the keywords have no direct relationship with other subjects in the cluster. This is a usual case in Co-word analyses (19). The hierarchical clustering by Co-word is shown in Figure 3.

### Strategic diagram

**Figure 4:**
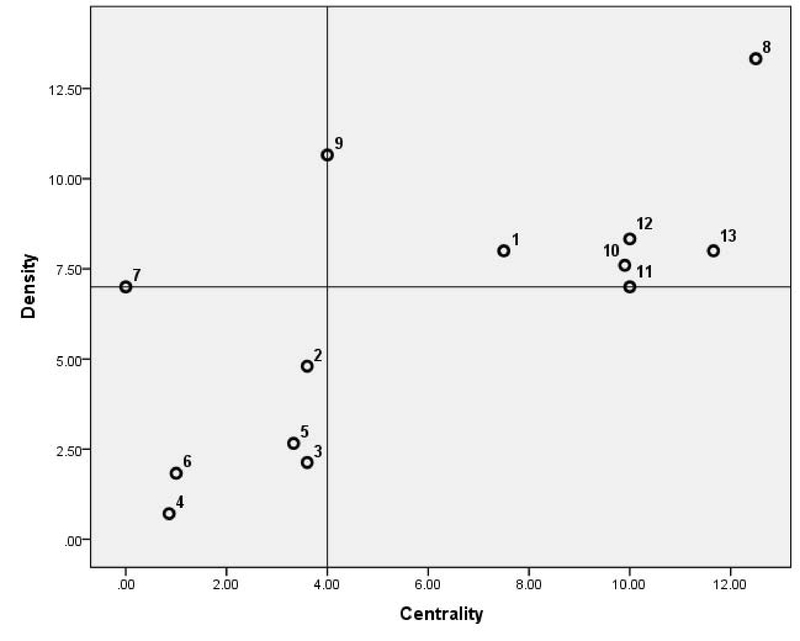
The strategic diagram of 13 clusters.

In this study, the centrality-density matrix was obtained by Ucinet 6.0. After that, a strategic diagram is drawn based on the centrality and density of 13 clusters (Table V and Figure 4). “A strategic diagram is mostly used to describe the internal relations within a cluster, as well as the interactions among different fields” (21). As shown in Table V, the clusters 8, 13 and 11, 12, respectively have higher centralities. It points that the clusters have joined well with other clusters of Andrology, and the clusters 7, 4; 6, 5; and 2, 3 have a lower centrality. These are considered as marginal clusters of Andrology. Based on co-word analysis in the field of Andrology, the strategic diagram of clusters was illustrated. Points 4 and 7 on the diagram shows the mean value of the centrality and the density of clusters. As already mentioned, the horizontal axis represents the centrality and determines the power of interaction of each cluster in the area under study. The vertical axis represents density and shows the internal relation in the subject of research. As the density of a cluster is higher, the cluster will have more potential for development (18). The seven clusters (1, 8, 10, 11, 12, 9, and 13) stood in part 2 of the strategic diagram. The density and centrality of these clusters are high. High density shows high internal correlation, while high centrality shows that these clusters are broadly joint to other clusters. Conversely, the six clusters (2, 3, 5, 6, 7, and 4) were placed in part 3 of the strategic diagram. This implies that these clusters are not axial, but are developing.

**Table 1:** Top five countries that contributed to producing scientific articles on Andrology over the period 2008-2017


**No.**	**Country**	**Frequency**	**Percentage**
1	USA	754	19.378
2	China	496	12.747
3	Italy	300	7.71
4	Iran	275	7.068
5	Germany	223	5.731

**Table 2:** Top five journals that contributed to producing scientific articles in Andrology over the period 2008-2017


**No.**	**Journal**	**Frequency**	**Percentage**
1	Fertility and sterility	358	9.201
2	Andrologia	258	6.631
3	Human reproduction	224	5.757
4	Journal of assisted reproduction and genetics	125	3.213
5	Asian journal of andrology	121	3.11

**Table 3:** The top 20 frequently used keywords in Andrology area over the period 2008-2017


**No.**	**Keywords**	**Frequency**	**No.**	**Keywords**	**Frequency**
1	Spermatozoa	333	11	Sperm injections, intracytoplasmic	122
2	Azoospermia	269	12	Testosterone	119
3	Varicocele	242	13	DNA damage	100
4	Semen analysis	237	14	Sperm motility	91
5	Semen	221	15	Polymorphism, genetic	88
6	Spermatogenesis	199	16	Fertilization in vitro	81
7	Testis	158	17	Y chromosome	69
8	Oligospermia	137	18	Reproductive techniques, assisted	63
9	Oxidative stress	134	19	Hypogonadism	62
10	DNA fragmentation	122	20	Obesity	62

**Table 4:** Frequency of the top Co-words


**Rank**	**Co-words**	**Frequency**
1	Azoospermia* oligospermia	51
2	Spermatozoa * semen	40
3	DNA fragmentation* spermatozoa	30
4	Spermatogenesis* testis	27
5	Sperm injections, intracytoplasmic* IVF	26
6	DNA damage* spermatozoa	26
7	Varicocele* semen	25
8	Oxidative stress* spermatozoa	25
9	Testis* spermatozoa	23
10	Testosterone* hypogonadism	21
11	Semen analysis* spermatozoa	19
12	Semen* oxidative stress	18
13	Y chromosome* azoospermia	18
14	Obesity*body mass index	15
15	Sperm motility* asthenozoospermia	14
16	Oligospermia* asthenozoospermia	9
17	IVF*reproductive techniques, assisted	9
18	Polymorphism, genetic* azoospermia	8
19	Reproductive techniques, assisted*semen analysis	5
20	Hypogonadism*metabolic syndrome	5

**Table 5:** Density and centrality of 13 clusters


**Cluster No. **	**Name of the clusters**	**Centrality**	**Density**
1	Body mass	7.50	8
2	Spermatogram	3.60	4.80
3	Genetics	3.60	2.13
4	Post-Testicular causes	0.86	0.71
5	Pre-Testicular causes	3.33	2.66
6	Idiopathic male infertility	1	1.83
7	Neoplasms	0	7
8	Reproductive techniques	12.50	13.33
9	Varicocele	4	10.66
10	Sperm biology	9.900	7.60
11	Sex steroids	10	7
12	Testicular-factor	10	8.33
13	Spermatogenesis	11.66	8

**Cluster 1: **Body mass: This cluster consists of three keywords, “obesity,” “Body Mass Index,” and “Sperm Quality” in a way that the existence of these three keywords reveals that the cluster deals with the relationship between body mass index and sperm parameters.

**Cluster 2**: Spermatozoa: This cluster consists of six keywords, “Sperm Motility,” “Sperm Count,” “Sperm Concentration,” “Sperm Morphology,” “Semen Analysis,” and “Smoking.” These keywords are important in andrology researches.

**Cluster 3**: Genetics: Six keywords have contributed to the formation of this cluster. Important keywords in this cluster, that are: “In Situ Hybridization Fluorescence,” “Aneuploidy,” “Meiosis,” “Y chromosome,” “Genetics,” and “Cryptorchidism”, indicate that the subject of this cluster can be genetics.

**Cluster 4**: Post-Testicular causes: The existence of keywords such as “Sperm Parameters,” “Reproduction,” and “Erectile Dysfunction” in this cluster represents the conditions that affect the male genital system after testicular sperm production.

**Cluster 5**: Pre-Testicular causes: This cluster has a significant semantic relationship with cluster 2. Five keywords are in this cluster and there are some important keywords like “Oligospermia,” “Azoospermia,” “polymorphism,” “Single Nucleotide” and “DNA Methylation,” which demonstrate that this cluster refers to conditions that impede adequate support of the testes and include situations of poor hormonal support and poor general health.

**Cluster 6**: Idiopathic male infertility: This cluster is closely related to cluster 3. The important keywords in this cluster are “Polymorphism, Genetic” “Receptors, Androgen.”

**Cluster 7**: Neoplasms: This cluster is related to “Cryopreservation” of testicular tissue to treat cancer.

**Cluster 8: **Reproductive Techniques: Three keywords (“IV",” “AR",” “ICSI”) in this cluster clearly show the techniques used to enhance fertility.

**Cluster 9:** Varicocele: In this cluster, the main subject is “Apoptosis,” “DNA Fragmentation,” and “Varicocele”, which is related to male genetic diseases.

**Cluster 10:** Sperm Biology: This cluster consists of six keywords. The keywords such as “DNA Damage,” “Oxidative Stress,” “Antioxidants,” “Human spermatozoa,” and “semen” in this cluster are related to Reproductive Biology from the Cellular and Molecular viewpoint. It refers to the complicated molecular mechanisms and the recognition of these mechanisms is worthwhile.

**Cluster 11:** Sex steroids: This cluster consists of four keywords and is located in the field of gonadal hormones. “Testosterone,” “Follicle-stimulating hormone,” and “Inhibin B” are the most important keywords in this cluster.

**Cluster 12:** Testicular-factor: The keywords like “Klinefelter Syndrome,” “Azoospermia,” “Sperm Retrieval,” and “Testicular Sperm Extraction” in this cluster suggest general subject of testicular factors.

**Cluster 13:** Spermatogenesis: This cluster has five keywords. “Spermatogenesis,” “Epididymis,” “Testis,” “Azoospermia, Nonobstructive” and “Spermatozoa” are important keywords from this cluster that are related to sperm production.

## 4. Discussion

In this study, Co-word analysis was conducted by using SPSS 20.0 and UcINEt 6.0 for obtaining a clear understanding of the growth of Andrology during the past 10 years. This study, by giving some reasonable results, identifies the factors such as the main research focus, the correlation between topics, and the current situation and trends in the field. The USA was the leading country in publication output on Andrology. This country in other biomedical fields has also been on top (10, 22, 23). About 34% of papers were published in five journals - Fertility and Sterility, Andrologia, Human Reproduction, Journal of Assisted Reproduction and Genetics, and Asian journal of Andrology. The most frequently used word throughout the periods was “Spermatozoa.” The keyword that has the most occurrences with this word was “semen.” “Sperm” is an essential part of “Male Fertility.” To date, the diagnosis of male infertility is commonly based on standard semen analysis. The male partner is considered a patient when an abnormality in semen parameters involving motility, morphology, or concentration has been detected in at least two semen analyses” (24). Therefore, it is no surprise “spermatozoa” was a hot keyword. “Azoospermia” and “Varicocele” are ranked second and third, respectively, among frequently used thematic in WoS over the research time span. Azoospermia is observed in 10-15% of infertile men (23, 25). “As the result, azoospermia has occurrence with “Oligospermia” which is related to “Male Infertility.” A reduced sperm density (oligozoospermia) is often accompanied by poor motility and morphology reflecting qualitative and quantitative defects in spermatogenesis” (26). “Varicocele” is a common genitourinary system disease, a condition that impairs production and decreases the quality of sperm, which was also a hot keyword (27)”. “Varicocele” is the most common cause of male infertility affecting about 15-20% of the general population and 35-40% of men presenting for an infertility evaluation” (23, 28).

“Many studies focused on “Semen Analysis,” which was the most prevalent technique during 1995- 2014” (23). Through the detailed analysis of a fresh semen sample, the semen can be determined by the health of the sperm. So the results of this analysis can help to identify problems and improve the fertility.

By analyzing the topics attributed to the documents (keywords), a wide range of scattered data was located in 13 clusters. The topics of these clusters are: “Body Mass,” “Spermatozoa,” “Genetics,” “Post-Testicular causes,” “Pre-Testicular causes,” “Idiopathic male infertility,” “Neoplasms,” “Reproductive Techniques,” “Varicocele,” “Sperm Biology,” “Sex steroids,” “Testicular-factor,” and “Spermatogenesis.”

The clusters created with common features within each group have structural relationships with each other and clusters represent a research director of the subject. The main axis of the subjects was “Spermatozoa.” “The diagnosis of male infertility has depended upon a descriptive evaluation of human semen with emphasis on the number of spermatozoa that are present in the ejaculate, their motility, and their morphology. The fundamental tenet underlying this approach is that male fertility can be defined by reference to a threshold concentration of motile, morphologically normal spermatozoa that must be exceeded in order to achieve conception” (29). The cluster analysis obtained in this study suggests that researchers emphasize genetic, anatomical, physiological and physical factors. This finding is consistent with other research (23, 30). Environmental and behavioral factors have not been considered.

As a result, the strategic diagram is employed to complement hierarchical clustering in the Co-word analysis. Clusters 8 and 13, “Reproductive Techniques” and “Spermatogenesis” in first quadrant are two most comprehensive subject areas, and that they are the mainstream issues in research on Andrology.

With regard to the therapy, “Intracytoplasmic sperm injection (ICSI),” which was one of the three highest ranking terms, appeared to be the main measure for treatment. “From the time Palermo and colleagues (1992) first used this technique, ICSI has been used more and more frequently” (23). “Since 2000, “gene polymorphism,” “DNA fragmentation” and “Apoptosis" emerged as core themes particularly between 2000 and 2004. It indicates that researchers understood that these variations or changes in the DNA sequence may significantly contribute to spermatogenesis and germ cell development, and thus have an impact on individual's response to certain drugs or even influence the risk of developing male infertility” (23). “ICSI,” “IVF” (i.e., in-vitro fertilization), and “assisted reproductive techniques” in recent years have been common treatments (16, 31, 32). Today, the assisted reproduction techniques are an effective method to treat the male factor infertility. This finding is consistent with other research (33, 16). “Spermatogenesis is the process by which haploid spermatozoa develop from germ cells in the seminiferous tubules of the testis. This process is absolutely necessary to fertilize the men. It is clear that accurate recognition of the state of spermatogenesis with the aim of finding a minimum number of spermatid or spermatozoa cells for the use of these cells according to reproductive techniques for oocyte fertilization is very important. Also, a better understanding of the biology and mechanisms involved in impaired spermatogenesis is important for the optimization of diagnostic and therapeutic management of both male and couple infertility” (34). The other themes that have high density and centrality include Sex steroids, “Testicular-factor,” “Sperm Biology,” and “Body Mass.” These subjects are the centers of Andrology. In other words, they are well-developed and have powerful internal correlation and maturation. Many clusters including Genetics, Post-Testicular causes, Pre-Testicular causes, Idiopathic Male Infertility, and Neoplasms are located in part 3 (Figure 4). Low density and centrality of them show that they are not highly associated with the topics in the other clusters Thus, they may involve emerging or disappearing topics. In another word, “genetic factors can be funded in major etiologic categories of male infertility (pre-testicular, testicular, and post-testicular forms), and genetic tests became part of the usual diagnostic procedure of patients” (35). This finding is consistent with other research (36, 37). It should be taken that the results of the research are based on WoS database. Using other databases such as PubMed and Scopus has probably different results.

## 5. Conclusion

This research reported a perspective on the intellectual structure of knowledge in Andrology studies during the period 2008-2017. Considering the frequency of keywords along with the clusters obtained, it seems that the most research approach was seen on infertility treatments, especially through assisted reproductive technology. In fact, infertility is a complicated life crisis which psychologically threatens and emotionally creates stress, and it is a considerable part of the research in the medical community due to the impact of infertility in human life. Despite the importance of psychological aspects as well as education of reproductive health, these subjects have not been sufficiently considered. Therefore, it is suggested that researchers focus on subjects that are marginal.In addition, the researchers can perform other techniques such as studying co-authorship and creating scientific maps.

##  Conflict of Interest

There is no conflict of interest about this paper.
